# Optimization of Image Quality in Pelvis Lymphoscintigraphy SPECT/CT Using Discovery NM/CT 670

**DOI:** 10.1055/s-0044-1790570

**Published:** 2024-09-26

**Authors:** Maryam Ghaneh, Shahrokh Nasseri, Ramin Sadeghi, Seyed Rasoul Zakavi, Habibeh Vosoughi, Mehdi Mommennezhad

**Affiliations:** 1 Department of Medical Physics, Faculty of Medicine, Mashhad University of Medical Sciences, Mashhad, Iran; 2 Nuclear Medicine Research Center, Mashhad University of Medical Sciences, Mashhad, Iran; 3 Research Center for Nuclear Medicine, Tehran University of Medical Sciences, Tehran, Iran

**Keywords:** lymphoscintigraphy, optimization, image quality, SPECT/CT, image reconstruction

## Abstract

**Aim** A lymphoscintigraphy is a crucial diagnostic tool for visualizing lymph nodes. This scan plays a significant role in determining the treatment and recovery plan for the patients. Due to the small lymph node size, obtaining high-quality images is important to prevent inaccurate results. We aimed to identify the most effective method for enhancing image quality through postprocessing techniques and altering the image reconstruction process.

**Methods** Two data sets were utilized in this study. First, National Electrical Manufacturers Association body phantom was filled with [ ^99m^ Tc]Tc-pertechnetate and prepared with and without any activity in the background of the body. Second, the images of 50 patients who underwent single-photon emission computed tomography/computed tomography imaging received [ ^99m^ Tc]Tc-phytate were collected. Discovery 670 GE gamma camera was used for imaging. Preprocessing of all images was performed by Xeleris and 3DSlicer 5.2.2 software was used for quantification. The effect of image reconstruction parameters such as resolution recovery (RR) algorithm, iteration, subsets, cutoff, and power in Butterworth filter, and full width at half maximum (FWHM) of Gaussian filter was assessed. The image quality index was determined based on contrast-to-noise ratio (CNR), contrast, and coefficient of variation.

**Results** The utilization of the RR algorithm showed notable improvements equal to 74, 35, and 38% of CNR, contrast, and noise reduction, respectively. Significant differences were observed in subiteration of 40 to 112 ( *p* -value < 0.05). The alteration of effective parameters in both smoothing filters yielded statistically significant results, leading to enhanced detectability, reduced noise, and improved contrast simultaneously. Optimum results in terms of noise reduction and CNR were achieved with subiteration (i × s) 4 × 12 using a Gaussian filter with FWHM of 4 or Butterworth filter with power of 10 and cutoff of 1. The highest contrast was observed at subiteration 40 using the Butterworth filter with cutoff of 0.5 and power of 5 or Gaussian filter with 2 mm FWHM. Qualitative analysis by two nuclear medicine specialists validated the quantified image quality.

**Conclusion** The reconstruction setting involving subiteration 48 with the Butterworth filter using cutoff of 1 and power of 10 or 4 mm FWHM of Gaussian filter produced the highest quality images.

## Introduction

Currently, around two-thirds of cancer cases are situated in the lower abdominal and pelvic regions. Cancer cells that metastasize often travel through the bloodstream and tend to settle in lymph nodes and channels. 1 2 Understanding the extent of involvement and pathways of lymph nodes is crucial for guiding surgical interventions. Accurate diagnosis through imaging and surgery plays a pivotal role in effective cancer treatment. 3 4 With the development of new tools and techniques, imaging procedures have been accompanied by significant progress, leading to better cancer diagnosis and prevention of additional surgery. 5 These methods are used to detect tumors and other abnormalities to diagnose the presence of disease and determine the effectiveness of treatment. Cancer that starts in the cervix can spread to the lymph nodes in the pelvis. 6 The most common method for examining lymph nodes before surgery is to use a lymphoscintigraphy scan. 7 Because of the small size of lymph nodes, the diagnosis of them in this scan is along with the false positive or false negative. 8 9 This scan can utilize single-photon emission computed tomography/computed tomography (SPECT/CT) to evaluate lymph nodes before surgery, 10 providing information on the level of involvement and the anatomical location of these. While this method generally has a low false negative rate, errors can still exist due to factors like attenuation, scattering, and noise, which can degrade image quality. 11 12 Lymphoscintigraphy may not show any lymphatic drainage in many patients. Although the false negative rate in the SPECT/CT technique is low, its probability is not zero. 13 14 Therefore, the images must have the highest quality to prevent errors and misdiagnosis. Tissue attenuation, scattering, and noise cause the quality of images to decrease. 15 16 17 Reconstruction methods affect the quality of images. The number of iteration, subset, and type of filter could alter the image quality. 18 Using the more subiterations and post-smoothing filter like Gaussian and Butterworth make the images smoother with lower detectability of small objects. Also, attenuation correction, scatter correction, and resolution recovery (RR) are often performed during image reconstruction to achieve higher quality.

The present study aimed to find the image reconstruction protocols that reduce the false negative or positive diagnosis in the SPECT/CT lymphoscintigraphy scan. All image reconstruction parameters were optimized using quantitative analysis and visual assessment.

## Methods

### Data Collection

The National Electrical Manufacturers Association image quality phantom containing six fillable spheres with diameters of 10, 13, 17, 22, 28, and 37 mm was used in the phantom study part of our research. 19 The spheres were filled with a homogenous [ ^99m^ Tc]Tc-pertechnetate solution at an activity concentration of 70 kBq/mL. Phantom imaging was acquired in two conditions, first, when the background of the body was air and the second when the background had been filled with about 7 KBq/mL of [99mTc]Tc-pertechnetate solution to obtain sphere-to-background ratio 10:1.

Fifty female patients, between 27 and 72 years, an average of 56 years, who underwent lymphoscintigraphy and SPECT/CT of pelvic, were selected. Seventy-five percent of them were postmenopausal. All patients suffered from malignancies affecting the uterus, ovaries, and cervix. Pelvic lymph node involvement was an important factor in choosing the patients and some parameters like age, weight, and stage of disease were neglected. The images of patients were retrospectively collected and retrieved from the hospital's picture archiving and communication system.

### Image Acquisition

Data acquisition of the phantom and patients was performed using the dual-head GE Healthcare Discovery NM/CT 670 CZT system with low-energy high-resolution collimators. Imaging parameters of CT images were 80 kV tube voltage, 50 mA tube current, 2.5 mm slice thickness, and 256 × 256 matrix size. SPECT imaging was acquired in 120 projections at 25 seconds per projection, step-and-shoot mode, and 128 × 128 matrix size.

### Image Reconstruction

**Fig. 1:**
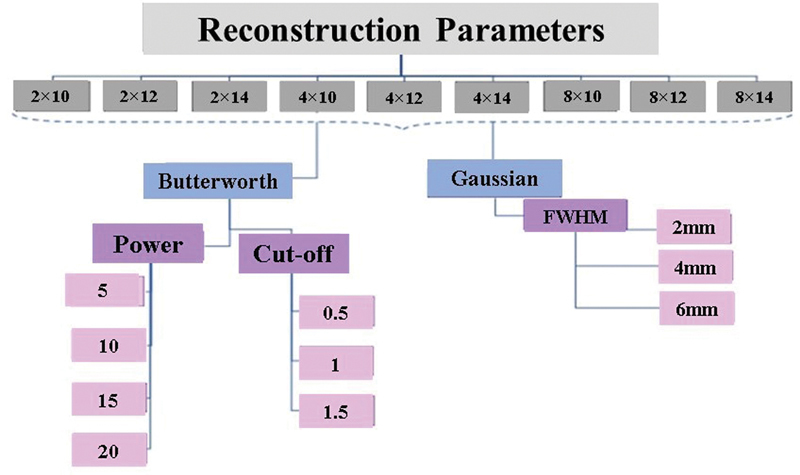
The flowchart of reconstruction parameters was used in this study. Reconstruction parameters include different subiterations with various full width at half maximum (FWHM) of Gaussian filter or using Butterworth filter with different power and cutoff. All reconstruction sets were repeated using the resolution recovery (RR) algorithm.

Vendor-provided Xeleris software was used for image reconstruction. According to Xeleris options, all raw data of SPECT images were reconstructed using the ordered subsets expectation-maximization (OSEM) algorithm with a combination of three iterations 2, 4, and 8, and subsets 10, 12, and 14. Gaussian filter with three different full width at half maximum (FWHM) 2, 4, and 6 mm and Butterworth filter with 0.5, 1, and 1.5 cutoff and power of 5, 10, 15, and 20 were separately applied on the images as the post-smoothing filters. Attenuation and scatter corrections were performed in all SPECT images. Additionally, all SPECT images were reconstructed once more applying the RR to assess the RR algorithm. In total, 270 SPECT images were obtained by applying different reconstruction settings. Quantitative and qualitative assessment of phantom images was carried out for the sphere with the 13-mm diameter because of the similarity of the size of lymphatic nodules in clinical situation. The reconstruction settings employed in our study are demonstrated in Fig. 1 .

### Image Preprocessing

Reconstructed images should be preprocessed before quantitative analysis. We created a three-dimensional (3D) model for the sphere's phantom segmentation using IGT module in 3DSlicer 5.2.2. The segment editor tool of 3DSlicer 5.2.2 was utilized for lymph node delineation in SPECT images. The volume of interest (VOI) of spheres and lymph nodes represents the cumulative activity in the selected region. The average and maximum count of each VOI, average count of background, and standard deviation of background count were measured using the quantification module and segment statistic tool in 3DSlicer 5.2.2.

### Quantitative and Qualitative Analysis

Three important metrics were calculated for evaluation of image quality in the sphere with 13 mm diameter of phantom and involved lymph node in patients; contrast-to-noise ratio (CNR), contrast, and noise (coefficient of variation [CV%]). CNR was calculated using Eq. (1) :



where *C* mean(sphere/nodule) is the average count in the region of interest (ROI) for the sphere in the phantom and nodule in the patient, *C* mean(background) is the average count of the spherical VOI in the background of the body phantom or the tissue surrounding the lymph node, and SD is the standard deviation of the background VOI. Contrast was computed with Eq. (2) :



where *C* max(sphere/nodule) is the maximum count in ROI, and *C* mean(background) is the average count of the spherical VOI in the background of the body phantom or the tissue surrounding the lymph node.

CV% as a statistical noise of image was calculated as follows Eq. (3) :



Two nuclear medicine specialists and one expert medical physicist visually evaluated the overall image quality and detectability of the lymphatic nodule in the pelvis and the sphere in the phantom. All images achieved from different reconstructions were qualitatively assessed and the best one was introduced.

### Statistical Analysis

All data were statistically analyzed using GraphPad Prism 10 software. CNR, contrast, and CV% obtained from all subiterations were compared using paired *t* -test analysis. After selecting the subiterations that showed significantly different from others, various parameters of the Butterworth (cutoff and power) and Gaussian (FWHM) filters were compared using the paired *t* -test in selected subiterations. The significance level of the *p* -value was set to less than 0.05 for all comparisons. The percentage difference of quantitative metrics was calculated to compare the role of the RR algorithm in image quality using Eq. (4) :



## Results

### Phantom

#### Quantitative Analysis

It is not feasible to calculate CNR and CV% when the body phantom background is devoid of any material, so only contrast can be calculated. Statistical analysis revealed a significant difference between subiterations 4 × 10, 4 × 12, and 4 × 14 in the calculation of CNR, contrast, and noise. The impact of the RR algorithm on the SPECT images was evaluated by the percentage difference of each index under two conditions; the presence of [ ^99m^ Tc]Tc-pertechnetate in the background and no radioactive material in the background of the body phantom.

Table 1 represents the image reconstruction sets most affected by the RR algorithm. Also, the percentage difference of calculated metrics for these reconstruction settings is demonstrated. RR algorithm improved CNR and contrast by 74% and more than 30%, respectively. Additionally, image noise considerably decreased by approximately 35% using the RR algorithm.

**Table 1:** Comparison of using the RR algorithm by calculating the percentage difference of image quality metrics in two SPECT images (with and without using the RR algorithm)

Background of body phantom	index	Image reconstruction set	Percentage difference
None	Contrast	2 × 14, Butterworth (power 20, cutoff 0.5)	+30
[ ^99m^ Tc]Tc-pertechnetate	CNR	4 × 10, Butterworth (power 20, cutoff 1)	+74
[ ^99m^ Tc]Tc-pertechnetate	Contrast	2 × 14, Gaussian (FWHM 4)	+40
[ ^99m^ Tc]Tc-pertechnetate	CV%	8 × 14, Butterworth (power 20, cutoff 1.5)	–35

Abbreviations: CNR, contrast-to-noise ratio; CV%, coefficient of variation; FWHM, full width at half maximum; RR resolution recovery; SPECT, single-photon emission computed tomography.

**Fig. 2:**
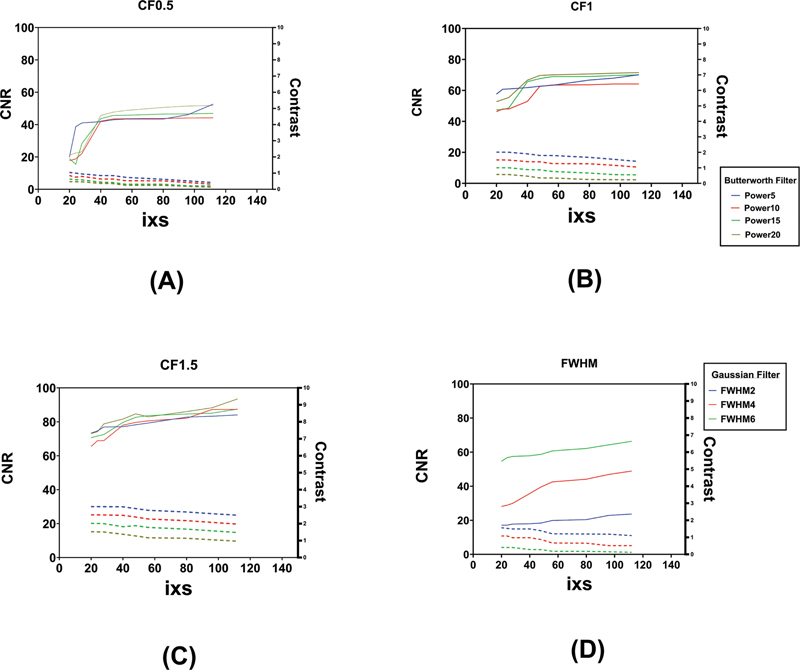
Contrast-to-noise ratio (CNR) and contrast tradeoff plots as a function of reconstruction sets. Dash lines represent CNR and dotted lines show contrast in all plots. ( **A** ) Represents all subiterations (i × s) with Butterworth filter using the cutoff of 0.5 and different powers, ( **B** ) represents all subiterations (i × s) with Butterworth filter using the cutoff of 1 and various powers, ( **C** ) demonstrates all subiterations (i × s) with Butterworth filter using the cutoff of 1.5 and different powers, and ( **D** ) shows all subiterations (i × s) with full width at half maximum (FWHM) 2, 4, and 6 mm of Gaussian filter.

Based on the results of scientific studies in this area, increasing CNR along with reducing contrast. 8 20 Comparisons of both metrics in the present study as a function of the reconstruction settings are shown in Fig. 2 . Regardless of the type of post-smoothing filter, subiterations 40 to 56 (4 × 10, 4 × 12, and 4 × 14 in our study) can produce high-quality SPECT images. As shown in Fig. 2A–C , while the Butterworth filter is applied, the appropriate parameters to achieve the best overall image quality are recommended to be 1 to 1.5 cutoff and 10 to 15 power. Also, according to Fig. 2D , a Gaussian filter with 4 mm FWHM seems suitable based on the tradeoff between CNR and contrast.

#### Qualitative Analysis

**Fig. 3:**
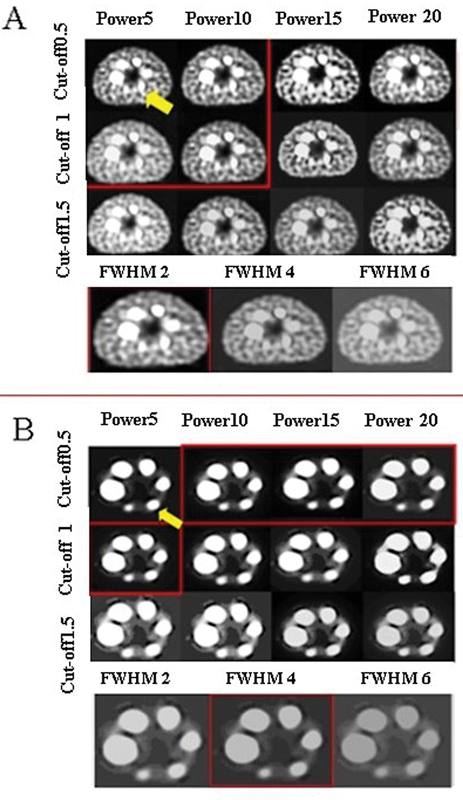
Transverse slices of National Electrical Manufacturers Association (NEMA) body phantom; ( **A** ) with filling [ ^99m^ Tc]Tc-pertechnetate in the background and ( **B** ) without any radioactive material in the background. The sphere with a 13-mm diameter is mentioned with yellow arrows. The selected reconstruction settings by the expert physicist and physicians are represented in red boxes.

Visual assessment was performed by a medical physicist and two nuclear medicine specialists. According to Fig. 3A , the most appropriate reconstruction setting was subiteration 4 × 12 with the Butterworth filter using cutoff of 0.5 or 1 and power of 5 and 10 in the presence of [99mTc]Tc-pertechnetate in the phantom background. Also, 2 mm FWHM of Gaussian filter can be used for smoothing. As shown in Fig. 3B , the best reconstruction set was subiteration 4 × 12 with Butterworth filter in the cutoff of 0.5 and 10, 15, and 20 of the power or cutoff of 1 and power of 5 in the phantom without any radioactive material in the background. However, the Gaussian filter with 4 mm FWHM was suitable too.

### Patients

#### Quantitative Analysis

The effect of the RR algorithm on the SPECT images was assessed by calculating the percentage difference of CNR, contrast, and CV%. Table 2 represents the image reconstruction settings most influenced by the RR algorithm. RR algorithm enhanced overall image quality resulting in a 42.2% reduction of noise, 74% CNR improvement, and 37.2% contrast enhancement.

**Table 2:** Comparison of using the RR algorithm by calculating the percentage difference of CNR, contrast, and, CV% in the SPECT images of patients

Index	Image reconstruction setting	Percentage difference
CNR	4 × 10, Butterworth (power 20, cutoff 1)	+74
Contrast	4 × 14, Butterworth (power 5, cutoff 1)	+37.2
CV%	8 × 14, Butterworth (power 10, cutoff 1.5)	–42.2

Abbreviations: CNR, contrast-to-noise ratio; CV%, coefficient of variation; RR resolution recovery; SPECT, single-photon emission computed tomography.

Statistical analysis showed a significant difference between four subiterations 4 × 10, 4 × 12, 8 × 10, and 8 × 14, and the other subiterations with *p* -values 0.0019, 0.022, 0.069, and ≤ 0.001, respectively. No significant differences were observed between the four mentioned subiterations.

**Fig. 4:**
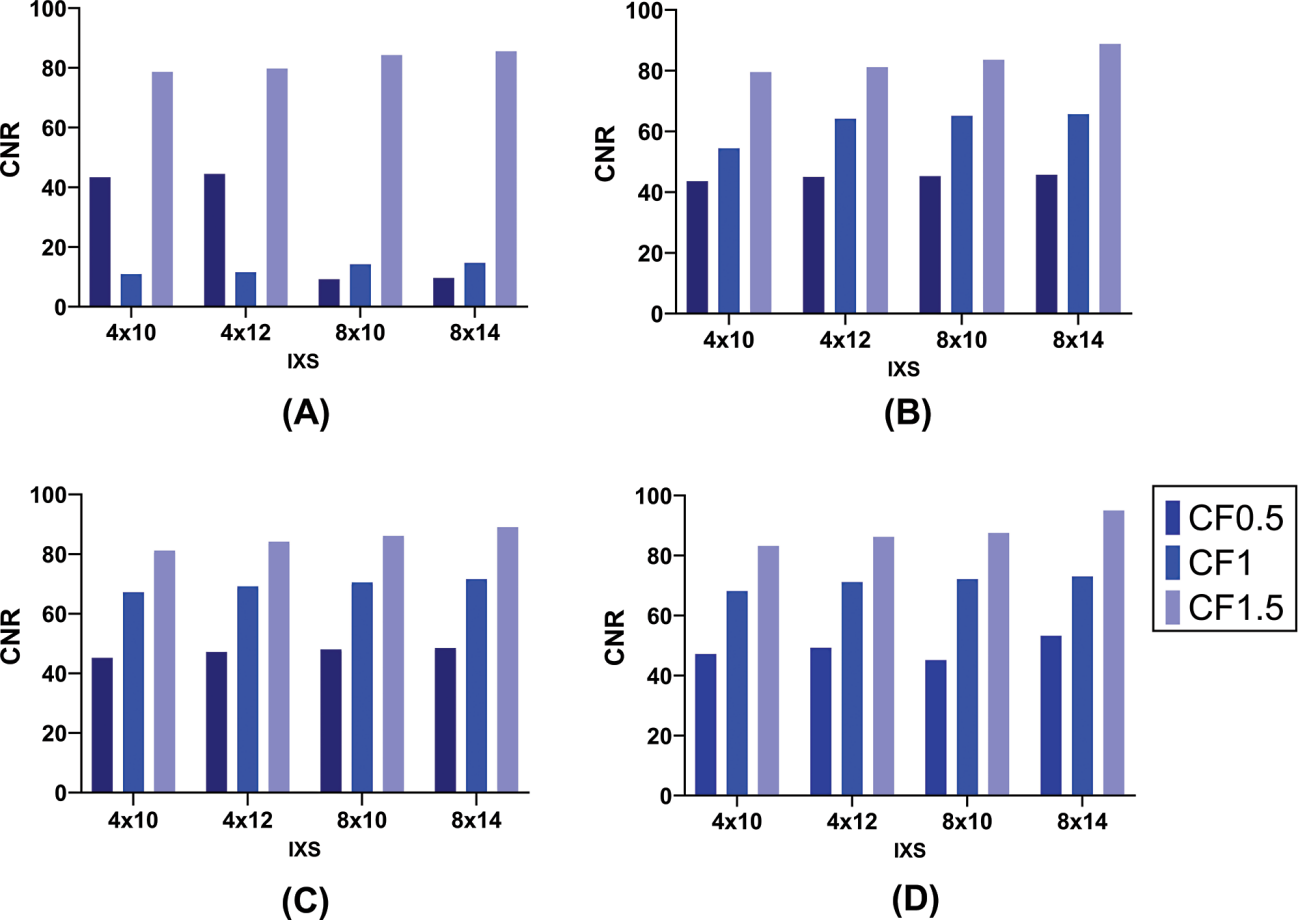
Bar chart plots of contrast-to-noise ratio (CNR) in different reconstruction settings. Comparison between four subiterations (4 × 10, 4 × 12, 8 × 10, and 8 × 14) using Butterworth filter with different cutoffs and ( **A** ): power of 5, ( **B** ): power of 10, ( **C** ): power of 15, and ( **D** ): power of 20. Each plot is related to an individual power and each cutoff is shown with a light to dark blue color.

As Fig. 4 shows, more subiterations create more CNR values. The highest CNR was obtained in power of 20 and cutoff of 1.5 by applying the Butterworth filter. Increasing the power and cutoff raises the CNR value in all subiterations. The difference between the lowest and the highest CNR in our investigated reconstruction sets was approximately 37%.

**Fig. 5:**
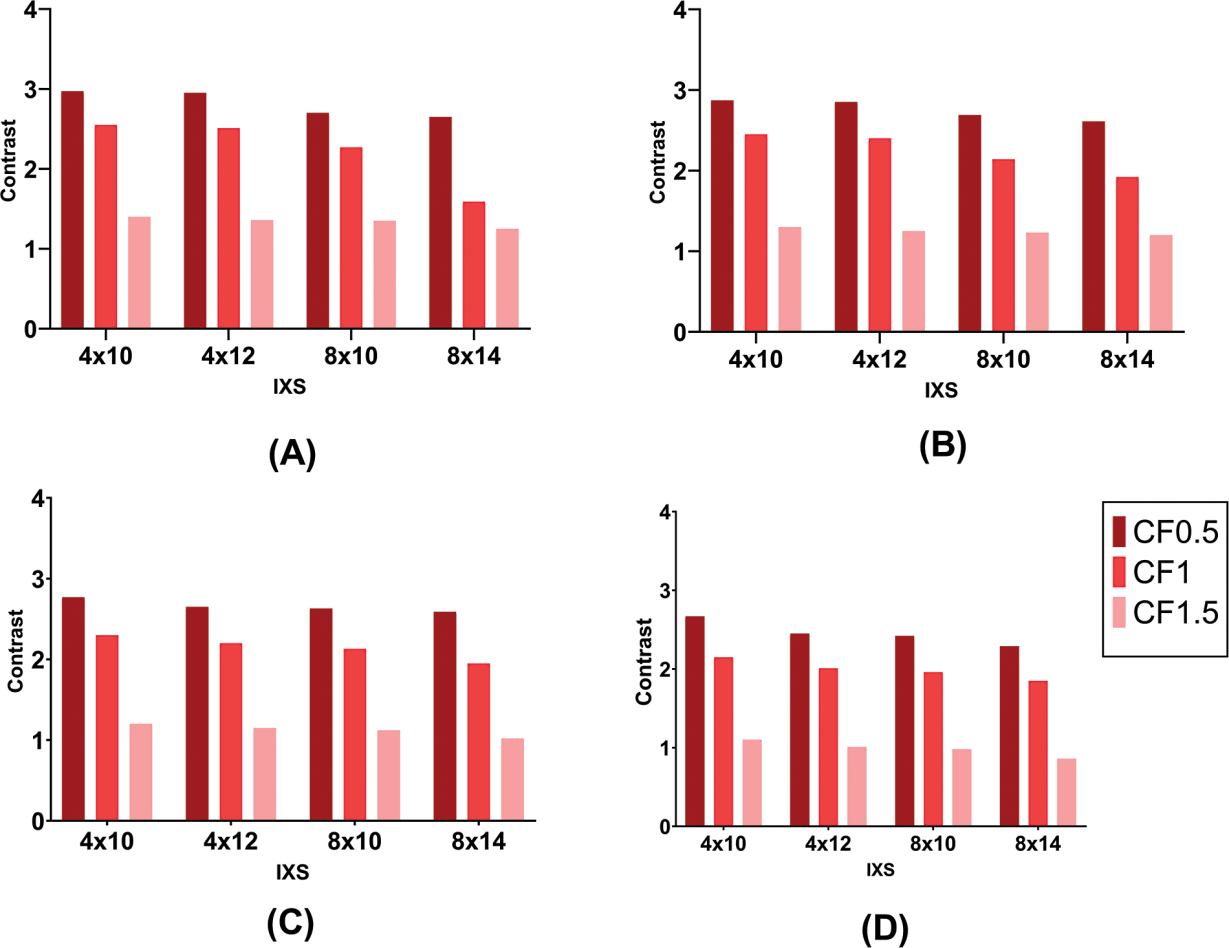
Bar chart plots of contrast in different reconstruction settings. Comparison between four subiterations (4 × 10, 4 × 12, 8 × 10, and 8 × 14) using Butterworth filter with different cutoffs and ( **A** ): power of 5, ( **B** ): power of 10, ( **C** ): power of 15, and ( **D** ): power of 20. Each plot is related to an individual power and each cutoff is shown with a light to dark red color.

According to Fig. 5 , more subiterations lead to less contrast values. The lowest contrast was obtained in the power of 20 and cutoff of 1.5 by applying the Butterworth filter. Increasing the power and cutoff could decrease the contrast value in all subiterations, albeit the role of cutoff is more considerable than power. The difference between the lowest and the highest contrast in our examined reconstruction sets was approximately 55%.

**Fig. 6:**
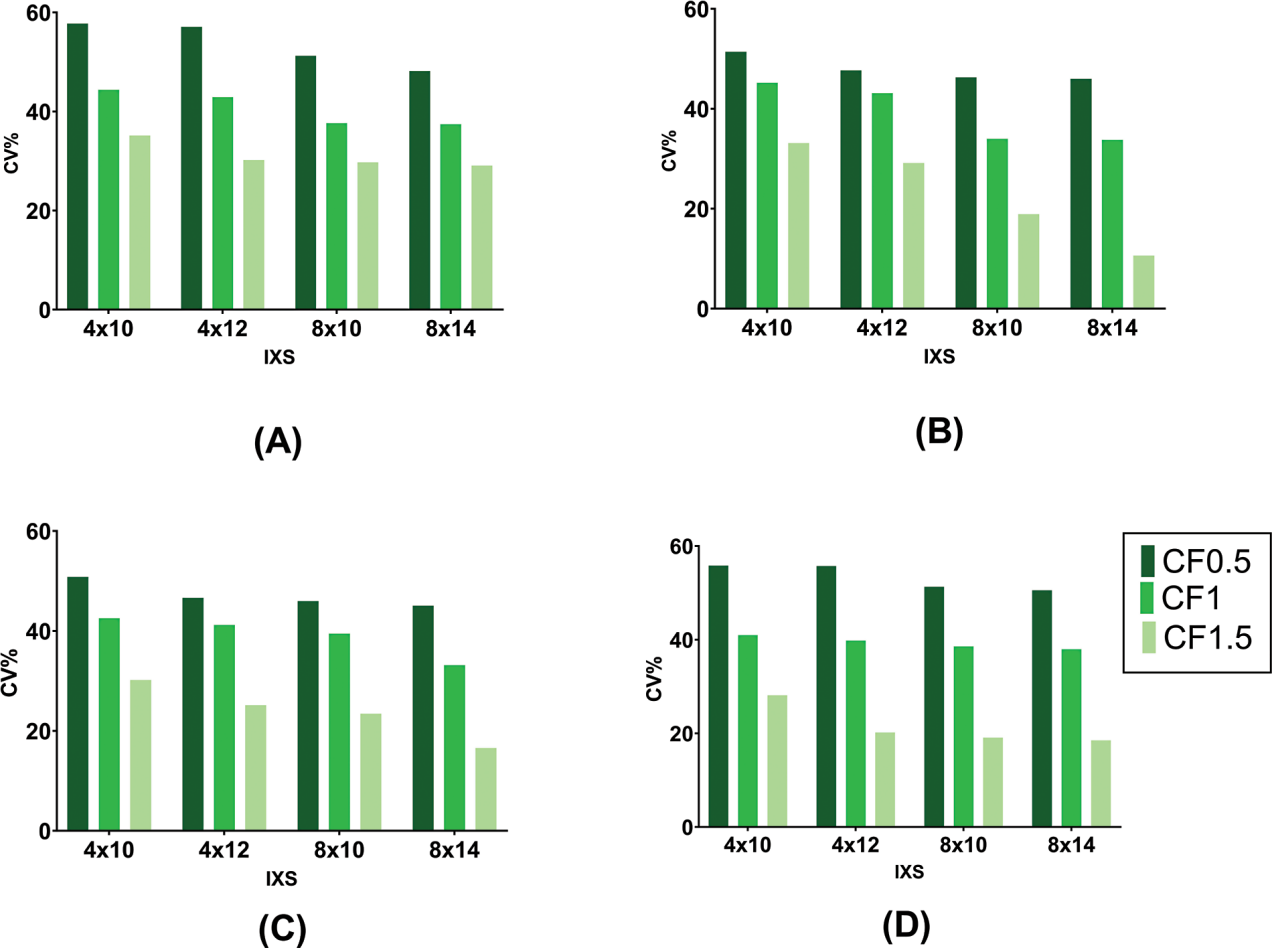
Bar chart plots of coefficient of variation (CV%) in different reconstruction settings. Comparison between four subiterations (4 × 10, 4 × 12, 8 × 10, and 8 × 14) using Butterworth filter with different cutoffs and ( **A** ): power of 5, ( **B** ): power of 10, ( **C** ): power of 15, and ( **D** ): power of 20. Each plot is related to an individual power and each cutoff is shown with a light to dark green color.

Fig. 6 illustrates the image noise reduced using more subiterations. The lowest CV% was obtained in subiteration 8 × 14 by applying the Butterworth filter with the power of 10 and cutoff of 1.5. The noise of the image could be reduced by approximately 51% if the appropriate cutoff has been selected.

**Fig. 7:**
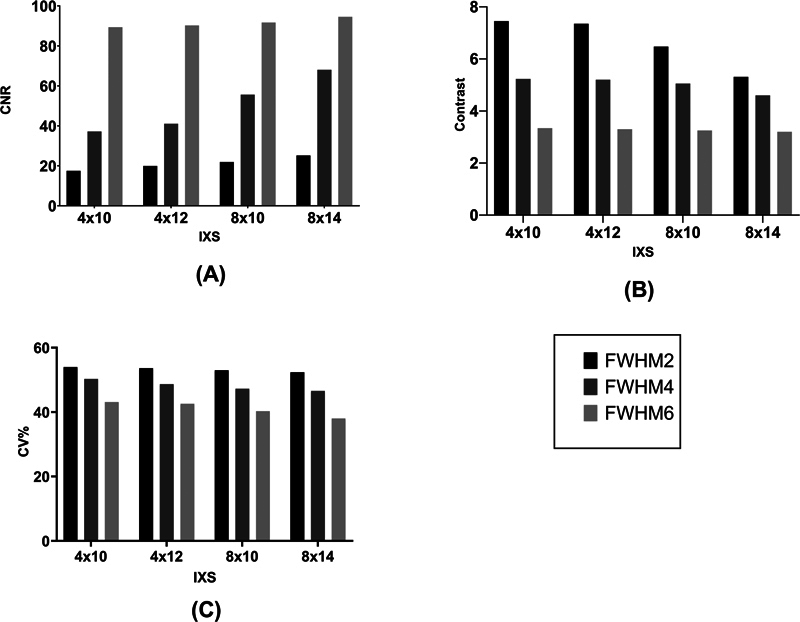
Comparison between four subiterations (4 × 10, 4 × 12, 8 × 10, and 8 × 14) using different full width at half maximum (FWHM) of Gaussian filter. Bar chart plots of contrast-to-noise ratio (CNR), contrast, and coefficient of variation (CV%) in different reconstruction settings are demonstrated in ( **A** ), ( **B** ), and ( **C** ) plots, respectively.

As shown in Fig. 7 , three quantitative metrics were evaluated in four subiterations with 2, 4, and 6 mm FWHM of the Gaussian filter. Increasing subiterations does not noticeably make a difference in the image noise, CNR, and contrast values, while FWHM of Gaussian filter impressively affects CNR and contrast. On average, CNR increases by 65%, contrast decreases by 36%, and noise reduces by 15% if 6 mm FWHM is used instead of 2 mm FWHM.

#### Qualitative Analysis

**Fig. 8:**
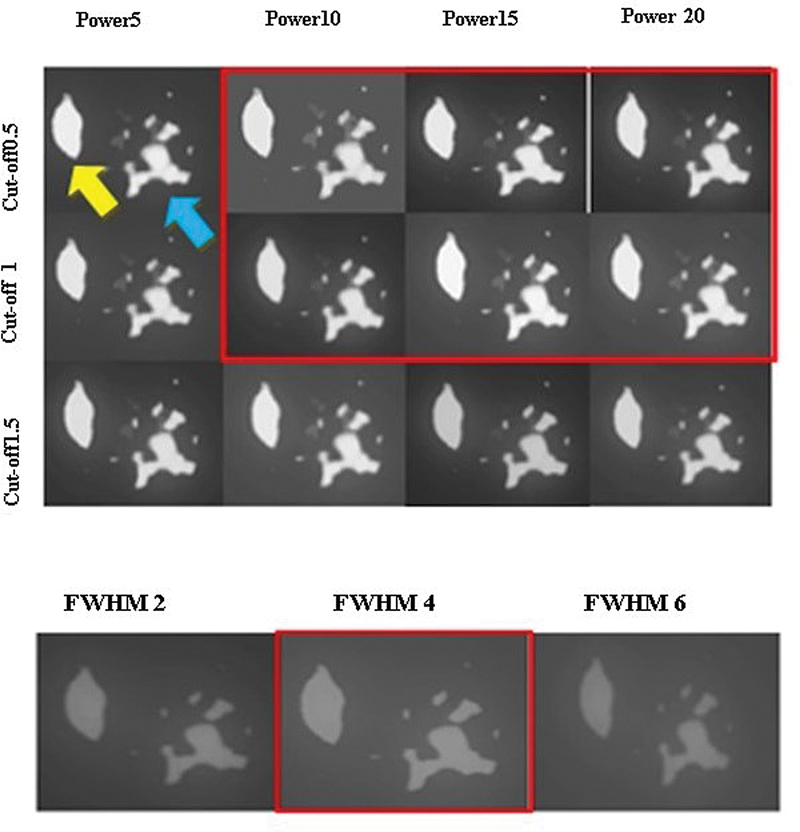
A transverse slice of one patient's reconstructed image using subiteration 4 × 12 with different parameters of Butterworth and Gaussian filters. The yellow arrow is the nodule and the blue arrow is the background in the surrounding tissue near the injection site. Preferred reconstruction sets by physicians are demonstrated in the red box.

Two nuclear medicine specialists clinically assessed images of all patients obtained from various reconstruction methods. The best image reconstruction protocol between four selected reconstruction sets was used for qualitative assessment. The transaxial images of one patient in different parameters of Butterworth or Gaussian filters in subiteration 4 × 12 are demonstrated in Fig. 8 . Preferred parameters by physicians in using the Butterworth filter tended to have less cutoff and more power values.

## Discussion

Generally, reconstruction sets using the high subiteration numbers, Gaussian filter with more FWHM and Butterworth filter with more cutoff and power, make images smoother. 18 Our findings show that CNR increased and image noise decreased by increasing the number of subiterations using smoother filters, while contrast is reduced in this situation. Also, overall image quality was improved using RR algorithm. RR algorithm could increase CNR and decrease contrast and noise almost 74, 40, and 35%, respectively ( Table 1 ). The effect of RR on reducing the imaging time and assessing image quality in cardiac acquisition was evaluated by Ismail and Mansor. He showed that RR gives better quantitative evaluation and results in a good resolution in the myocardial perfusion images. 21 Another study in 2021 demonstrated that using attenuation and scatter correction and RR algorithm in the OSEM reconstruction method could noticeably enhance spatial resolution in SPECT/CT of a Jaszczak phantom. 22 A retrospective study in 2022 showed RR improves spatial resolution and CNR in the bone SPECT images. 23

According to the inverse relationship between CNR and contrast, the higher the CNR the lower the contrast. This results in a loss of detectability especially in small objects. Therefore, the tradeoff between CNR and contrast in the phantom images filled with [ ^99m^ Tc]Tc-pertechnetate in the background that demonstrated the most appropriate reconstruction setting is sub-iteration 4 × 12 using Butterworth filter with cutoff of 1 and power of 10 or Gaussian filter with 2 or 4 mm FWHM ( Fig. 2 ). Based on our quantitative and qualitative analysis, it seems that the appropriate image reconstruction setting be subiteration 4 × 12 using Butterworth filter with cutoff of 0.5 and power of 5/10 or cutoff of 1 and power of 5/10 or 2 mm FWHM of Gaussian filter in the phantom with radioactive material in the background. Also, the appropriate image reconstruction setting in phantom without any radioactivity in the background is subiteration 4 × 12 using the Butterworth filter with cutoff of 0.5 and power of 10/15/20 or cutoff of 1 and power of 5 or 4 mm FWHM of Gaussian filter. As many research papers demonstrate, increasing the number of iterations, subsets, FWHM of Gaussian filter, and the more cutoff and power of Butterworth filter made higher CNR but lower contrast and noise. 24 25 26 Increasing the iteration numbers, cutoff frequency in the Butterworth filter, and reducing FWHM of the Gaussian filter leads to enhanced spatial resolution in the SPECT of a Jaszczak phantom. 22 Fukami et al optimized the number of iterations in SPECT images of thoracic spine phantom. They evaluated contrast, noise, and standardized uptake value as a quantitative analysis. Their results showed that increasing iteration numbers caused an increase in CV% and contrast. However, contrast almost converged uniformly in subiterations of more than 50. 27 The difference between this study and our work was the type of phantom used and the data analysis methods.

The proposed image reconstruction parameters based on the evaluation of patient images were subiteration 4 × 12, 4 mm FWHM of Gaussian filter, and Butterworth filter with cutoff of 1 and power of 10/15. Our finding is in agreement with the results of a study by Lanfranchi et al that recommended the optimized image reconstruction setting for SPECT of brain Dat-scan. They applied the Butterworth with a 0.96 cutoff. 9 Another study by Lyra and Ploussi on SPECT of the liver showed that the Butterworth filter with the cutoff of 0.1 to 0.5 could help to achieve the high detectability of small lesions. 28 We found the cutoff of 0.5 in the Butterworth filter as the optimized parameter in the reconstruction protocol, similar to the Lyra and Ploussi research. Two studies utilized the SPECT/CT images of cardiac 10 and bone scan. 14 In the myocardial perfusion scan, Gaussian filter with 12 to 14 mm FWHM was applied to smooth the images. 10 Optimized filters for the assessment of bony metastatic lesions were proposed 10 to 13 mm FWHM of Gaussian filter by Alqahtani et al. 14 These results were in contrast with our findings because of the object sizes. Extra smoothing of the images could lead to misdiagnosis and false negatives when the lesion size is small, like lymph nodes. While in large lesions such as obvious bone metastasis or cardiac muscle, more smoothing makes CNR better, reduces noise, and improves image quality without any false negativity. According to our results, 2 and 4 mm FWHM of Gaussian filter were recommended in lymphoscintigraphy SPECT/CT of pelvic. Choosing the appropriate reconstruction parameters strongly depends on the size of the lesions. Overall image quality does not degrade using the smoother filter in evaluating large objects, while it is influenced on detectability and assessment of small lesions.

Although many studies about the optimization of image reconstruction settings have been performed, none of them simultaneously assessed all parameters. We evaluated iterations, subsets, two different smoothing filters, and the RR algorithm to find the optimized reconstruction sets for small lesions. Nonetheless, our study had some limitations. First, we studied the optimization of reconstruction sets only for patients with lymph nodes involved in pelvic and these results cannot be generalized to other cases with different sizes of lesions. Second, we optimized the reconstruction parameter options that exist in one make and model of gamma camera scanner (GE Discovery 670 CZT), while it can be evaluated for other gamma cameras as a multicenter study similar to harmonization in positron emission tomography/CT scanner. 29 30

## Conclusion

According to our findings, subiteration 4 × 12 proposed with the Butterworth filter using a power of 10 and a cutoff of 1 or 4 mm FWHM of the Gaussian filter. Furthermore, utilizing the RR algorithm in the Discovery NM/CT 670 GE gamma camera is recommended to achieve the highest image quality in lymphoscintigraphy scans.
